# Different dietary patterns and reduction of lung cancer risk: A large case-control study in the U.S.

**DOI:** 10.1038/srep26760

**Published:** 2016-05-27

**Authors:** Huakang Tu, John V. Heymach, Chi-Pang Wen, Yuanqing Ye, Jeanne A. Pierzynski, Jack A. Roth, Xifeng Wu

**Affiliations:** 1 Department of Epidemiology, The University of Texas MD Anderson Cancer Center, Houston, Texas, USA; 2 Department of Thoracic/Head and Neck Medical Oncology, The University of Texas MD Anderson Cancer Center, Houston, Texas, USA; 3 Institute of Population Health Science, National Health Research Institutes, Zhunan, Taiwan; 4 Department of Thoracic and Cardiovascular Surgery, The University of Texas MD Anderson Cancer Center, Houston, Texas, USA.

## Abstract

Reducing lung cancer risk by modifying diet is highly desirable. We investigated whether different U.S. dietary patterns were associated with lung cancer risk. Dietary patterns were derived using exploratory factor analysis for 2139 non-small cell lung cancer (NSCLC) cases and 2163 frequency-matched controls. Logistic regression was used to estimate odds ratios (ORs) and 95% confidence intervals (95% CIs). Highest adherence (highest *vs*. lowest quintile) to the “Tex-Mex”, “fruits and vegetables”, and “American/Western” patterns was associated with a 55% reduced (OR = 0.45; 95% CI = 0.37–0.56; *P* < 0.001), 32% reduced (OR = 0.68; 95% CI = 0.55–0.85; *P* = 0.001), and 45% increased (OR = 1.45; 95% CI = 1.18–1.78; *P* < 0.001) risk of lung cancer, respectively. The effects were stronger for squamous cell carcinoma and ever smokers for the “fruits and vegetables” pattern, and stronger for other non-small cell lung cancer and never smokers for the “American/Western” pattern. Among six genome-wide association (GWA) studies-identified lung cancer susceptibility loci assessed, a variant (rs2808630) of the C-reactive protein gene modified the associations for the “fruits and vegetables” (*P* for interaction = 0.03) and “American/Western” (*P* for interaction = 0.02) patterns. Our study first showed that the “Tex-Mex” dietary pattern was associated with a reduced lung cancer risk. Also, the “fruits and vegetables” and “American/Western” patterns affected lung cancer risk, and the effects were further modified by host genetic background.

Lung cancer is the second most common cancer in both men and women in the U.S.^1^. Smoking is the most important risk factor for lung cancer^1^. In addition to smoking, other factors such as diet may also play a role in lung carcinogenesis^2^. Previous studies suggested that consumption of fruits as well as foods containing carotenoids probably decreased lung cancer risk^2^; however evidence on other dietary factors is not conclusive^2^. Typically, previous studies have focused on the effects of specific nutrients, but it is difficult to discriminate the effect of a specific nutrient due to the strong correlations between nutrients.

Dietary pattern analysis (e.g., factor analysis) is a novel way to examine the effect of diet on cancer^3^. A few studies have investigated the associations between dietary patterns and lung cancer risk using factor analysis^4^,^5^,^6^,^7^,^8^. Though the findings are not definitive, they all suggest that a “healthy” diet characterized by high vegetable intake is associated with a decreased risk of lung cancer, while a “Western” diet characterized by high fat and red meat intake is associated with an increased risk. However, most of the studies were conducted outside of the U.S.^5^,^6^,^7^,^8^, except one small study^4^. Also, evidence on other American dietary patterns is very limited. For example, Texas Mexican cuisine (“Tex-Mex”) is an American regional cuisine which is the most popular in the state of Texas and spreads to the rest of the U.S. as well as Canada.

Several studies showed that the associations of dietary patterns and lung cancer could be modified by host smoking status^4^,^5^,^6^,^9^,^10^. Due to the complex gene-diet interaction, the association of dietary patterns and lung cancer risk may be modified by host genetic background, which has not been investigated in previous studies. Previous genome-wide association (GWA) studies have identified multiple lung cancer susceptibility loci among individuals of European ancestry^11^,^12^,^13^,^14^,^15^,^16^, and two of the identified loci are mapped to the C-reactive protein (*CRP*) gene and interleukin 1 receptor accessory protein (*IL1RAP*) gene, respectively. Both genes play an important role in host inflammatory response which is closely linked to lung cancer development^17^. Since dietary factors are known to have a major effect on host inflammatory response^18^,^19^, it is likely that the associations of dietary patterns with lung cancer risk will be modified by these inflammation-related loci.

In the present study, we investigated the associations between three dietary patterns derived by factor analysis (“fruits and vegetables”, “American/Western”, and “Tex-Mex”) and non-small cell lung cancer (NSCLC) risk using data from a large ongoing Texas-based case-control study. Further, we investigated whether the associations differed by major histological types of NSCLC and whether the associations could be modified by host smoking status and lung cancer susceptibility loci identified in previous GWA studies.

## Results

### Identified dietary patterns and host characteristics

Exploratory factor analysis identified three dietary patterns that together accounted for 26% of the total variance, and the top 10 food items/groups contributing to each factor are listed in Table 1. The three dietary patterns were named “fruits and vegetables”, “American/Western”, and “Tex-Mex” based on the food items/groups that were strongly correlated with each dietary pattern. The spearman correlation coefficients of the factor scores for the three dietary patterns with nutrient intake are shown in Supplementary Table 1.

Selected characteristics of the 2139 NSCLC cases and 2163 controls are presented in Table 2. Cases and controls differed in education, smoking pack-year, family history of lung cancer among first degree relatives, body mass index (BMI), physical activity, and daily intake of protein.

### Associations of dietary patterns with non-small cell lung cancer risk

Age- and sex-adjusted and multivariable-adjusted associations of dietary patterns with lung cancer risk are presented in Table 3. In age- and sex-adjusted models, all three dietary patterns were associated with lung cancer risk (*P* for trend < 0.001). In multivariable-adjusted models, compared to the lowest quintile of the score on the “fruits and vegetables” pattern, the highest quintile was associated with a 32% decreased risk (OR_Q5 *vs*. Q1_ = 0.68; 95% CI = 0.55–0.85; *P* for trend = 0.001). Higher adherence to the “American/Western” dietary pattern was associated with an increased risk of lung cancer (OR_Q5 *vs*. Q1_ = 1.45; 95% CI = 1.18–1.78; *P* for trend < 0.001). Higher adherence to the “Tex-Mex” pattern was associated with a decreased risk (OR_Q5 *vs*. Q1_ = 0.45; 95% CI = 0.37–0.56; *P* for trend < 0.001).

### Stratified associations by histological types of non-small cell lung cancer and smoking status

The stratified associations by major histological type of NSCLC and smoking status are summarized in Tables 4 and 5, respectively. The three dietary patterns were associated with risks of all three major histological types. The protective effects of the “fruits and vegetables” pattern were more evident for squamous cell carcinoma. The harmful effects of the “American/Western” pattern were more pronounced for other NSCLC. The effects of the “Tex-Mex” pattern were similar across all histological types.

The negative association of the “fruits and vegetables” pattern with lung cancer risk was present among current or former smokers, and not present among never smokers, and the *P* for interaction was 0.03. The “American/Western” pattern was associated with an increased risk of lung cancer irrespective of smoking status; however, the association was stronger among never smokers, although the *P* for interaction (0.44) was not statistically significant. The association for the “Tex-Mex” pattern did not differ by smoking status (*P* for interaction = 0.87).

### Stratified associations by GWA studies-identified susceptibility loci

The overall associations between dietary patterns and lung cancer risk in this subset of study population were similar to these in the total study population (Table 3). Among the six selected SNPs, four (rs1051730, rs2808630, rs7626795, rs6495309) were associated with lung cancer risk in this sample (*P* < 0.05). The stratified associations of dietary patterns with lung cancer risk by genotype at rs2808630 of the *CRP* gene are summarized in Table 6. The “fruits and vegetables” pattern was associated with a reduced risk of lung cancer only among those without a copy of the minor allele (OR_Q5 *vs*. Q1_ = 0.42; 95% CI = 0.26-0.69; *P* for trend = 0.001; *P* for interaction = 0.03). In contrast, the “American/Western” pattern was associated with an increased risk of lung cancer only among those with at least one copy of the minor allele (OR_Q5 *vs*. Q1_ = 1.93; 95% CI = 1.27–2.93; *P* for trend = 0.001; *P* for interaction = 0.02). The “Tex-Mex” pattern was associated with a reduced risk of lung cancer irrespective of the genotype at *CRP* rs2808630 (*P* for interaction = 0.27). No statistically significant interactions (*P* for interaction > 0.05) were found between dietary patterns and the other five (rs1051730, rs3117582, rs7626795, rs402710, rs6495309) selected variants (Supplementary Table 2).

## Discussion

In this large Texas-based case-control study, we identified three dietary patterns using factor analysis: “fruits and vegetables”, “American/Western”, and “Tex-Mex”. Our study is the first to show that the “Tex-Mex” pattern was associated with a substantially reduced lung cancer risk. In addition, we found that the “fruits and vegetables” pattern was associated with a reduced risk and the protective effects were more evident for squamous cell carcinoma and among ever smokers. In contrast, the “American/Western” pattern was associated with an increased risk and the harmful effects were more pronounced for other NSCLC and among never smokers. Finally, for the first time, we found that the effects of the “fruits and vegetables” and “American/Western” patterns were further modified by a variant (rs2808630) of the *CRP* gene.

Our study is the first report to show that the “Tex-Mex” dietary pattern is associated with substantially reduced lung cancer risk, and the effects are consistent and stable across different sub-groups. Except our previous study with a smaller sample size which reported a non-statistically significant protective effect on renal cell carcinoma^20^, there has been no other studies on the “Tex-Mex” dietary pattern and cancer. The mechanism(s) linking high adherence to the “Tex-Mex” pattern with a decreased risk of lung cancer is unclear. “Tex-Mex” cuisine is characterized by its heavy use of legumes, spices, and shredded cheese. Legumes are rich sources of dietary fiber, a variety of micronutrients, and phytoestrogens with potential cancer-preventive effects^21^. In particular, our previous study showed that higher intake of phytoestrogens was associated with a decreased risk of lung cancer^22^. Also, spices or their bioactive components may prevent cancer through their anti-microbial, anti-oxidant, and inhibition of carcinogen bioactivation effects^23^. Finally, high levels of cheese intake were found to be associated with a reduction in lung cancer risk in multiple studies^24^,^25^,^26^, and menaquinones and conjugated dienoic derivatives of linoleic acid in cheese were suspected to mediate the protective effects^27^,^28^.

Our findings on the “fruits and vegetables” and “American/Western” dietary patterns are consistent with findings from previous studies on dietary pattern and lung cancer. Previous studies using factor analysis^4^,^5^,^6^,^7^,^8^ found that a “healthy” diet characterized by high vegetable intake was associated with a decreased risk of lung cancer, while a “Western” diet characterized by high fat and red meat intake was associated with increased risk. In addition to dietary patterns derived from factor analysis, several studies also investigated index-based dietary patterns. Three studies found that diet quality index was inversely associated with subsequent lung cancer risk^10^,^29^,^30^, and diet quality was assessed by the recommended foods score or dietary guideline index, which reflects compliance with the current dietary guidance of increasing consumption of fruits, vegetables, whole grains, lean meats or meat alternatives, and low-fat dairy. Additionally, a Mediterranean dietary pattern was found to be inversely associated with lung cancer risk^9^.

In our study, we found that the inverse associations of the “fruits and vegetables” pattern with lung cancer risk were only present among current or former smokers but not present among never smokers. This observation was consistent with the findings from previous dietary pattern studies on lung cancer that the beneficial effects of dietary patterns characterized by high consumption of fruits or vegetables were only evident in current or former smokers^4^,^5^,^6^,^9^,^10^. Smoking causes lung cancer in part through its pro-oxidant properties^31^. It is believed that the protective effects of fruits and vegetables are due to their rich collection of various antioxidants^32^,^33^,^34^, and this may explain why the protective effects were only seen in ever smokers. In addition, this is in line with our observation that the protective effects of the “fruits and vegetables” pattern were more evident for squamous cell carcinoma, which is most strongly associated with smoking among the main histological types of NSCLC^35^.

For the first time, we found that the “fruits and vegetables” and “American/Western” patterns interacted with a lung cancer susceptibility locus (rs2808630). The rs2808630 locus is mapped to the 3′ untranslated region of the *CRP* gene, which is a key gene in host inflammatory response^17^. Previous studies showed that higher fruit and vegetable intake was associated with lower circulating CRP levels^36^,^37^. Also, it is known that the “American/Western” diet leads to increased systemic inflammation^19^,^38^,^39^,^40^. According to a recent meta-analysis of 10 prospective studies^41^, circulating CRP levels, a marker for systemic inflammation, were positively associated with lung cancer risk. The *CRP* rs2808630 polymorphism has been shown to affect circulating CRP levels^42^,^43^. More importantly, one study showed that the GG genotype at *CRP* rs2808630, compared with the AA or AG genotype, was associated with a larger CRP increase in response to pro-inflationary stimuli, and this may explain our observation that the harmful effects of the “American/Western” pattern were only evident among those with the GG or AG genotype.

Our study has several limitations. First, the dietary data were collected for the year before diagnosis (cases) or enrollment (controls), and it may not represent the time window of interest (e.g., many years prior to lung cancer diagnosis when lung cancer has not been initiated yet). However, longitudinal studies showed that a single food frequency questionnaire measurement at one time point could characterize dietary habits for a period of at least 5–10 years^44^, and dietary patterns assessed with a food-frequency questionnaire were stable over time^45^. Second, our study is subject to recall bias because cases and controls may recall dietary intakes differently. Nevertheless, the direction and magnitude of the associations for the “fruits and vegetables” and “American/Western” patterns in our study were consistent with and comparable to those found in a prospective study where dietary data were collected around ten years prior to diagnosis^5^. Since the impact of the “Tex-Mex” dietary pattern on human health was not reported and therefore not publicized before, the findings for the “Tex-Mex” dietary pattern are less likely to be biased due to differential recall. Third, our analysis was limited to non-Hispanic whites, and caution should be taken when generalizing our results to other populations.

Despite these limitations, our study has several strengths. First, to our knowledge, this study is the first to show a protective effect of the “Tex-Mex” dietary pattern on lung cancer and the first to assess the interaction between dietary patterns and genetic variations on lung cancer risk. Second, our study had the largest sample size in terms of number of cases among the studies on dietary pattern and lung cancer risk using factor analysis. Third, the FFQ used in this study was previously validated. Finally, the two dietary patterns that explained the most variation in our study have been consistently identified in previous studies^4^,^5^,^6^,^7^,^8^, and we were able to assess a third dietary pattern (the “Tex-Mex” pattern) because consumption of “Tex-Mex” foods is relatively common in this Texas-based case-control study.

In summary, our study adds to the growing evidence that diet plays an important role in lung carcinogenesis which is thought by many to be caused solely by smoking. In particular, our study suggests that “Tex-Mex” cuisine may reduce lung cancer risk, and more studies are needed to confirm this novel finding and to explore the underlying mechanism(s). Also, our study together with previous studies supports that maintaining a “healthy” diet (increasing consumption of fruits and vegetables and limiting energy-dense and processed foods) may prevent lung cancer, and the beneficial effects are further modified by genetic background.

## Methods

### Study population

Cases and frequency-matched controls were accrued from a large ongoing case-control study of lung cancer. Cases were newly-diagnosed and histologically confirmed NSCLC patients from The University of Texas MD Anderson Cancer Center. There were no restrictions on age, sex, race/ethnicity, or stage. Healthy controls without a history of cancer (except for nonmelanoma skin cancer) were recruited from the Kelsey-Seybold Clinics, the largest private multispecialty physician group in the Houston metropolitan area with 18 clinics, more than 325 physicians and over 400,000 patients. The rationale of recruiting controls from the Kelsey-Seybold Clinic has been previously discussed^46^. When potential control participants visited the Kelsey-Seybold Clinic for annual physical exams, Kelsey-Seybold Clinic staff distributed a brief questionnaire to elicit the patients’ willingness to be contacted by staff at MD Anderson and to collect preliminary demographic data for frequency-matching. For those who were willing to participate, staff at MD Anderson then contacted them by telephone to confirm their willingness to participate and to schedule an in-person interview at a Kelsey-Seybold Clinic convenient to the participant. Controls were frequency-matched on age (±5 years), sex, race/ethnicity, and smoking status (current, former, never). To date, the response rate among both cases and controls has been approximately 80%. All participants provided written informed consent prior to participation in the study. This study was approved by The University of Texas MD Anderson Cancer Center and Kelsey-Seybold institutional review boards, and all methods and analyses were conducted in accordance with this approval.

### Data collection

MD Anderson staff interviewers conducted interviews to collect epidemiological data on demographics, education, smoking, family history of cancer, height, weight, and physical activity. Additionally, dietary intake during the year prior to diagnosis (cases) or study enrollment (controls) was assessed with a previously validated food frequency questionnaire (FFQ, a modified version of the National Cancer Institute’s Health Habits and History Questionnaire)^47^. The questionnaire has been shown to be a valid and reliable food frequency survey tool across various populations^48^,^49^. This questionnaire asks about the frequency and portion size of food and beverage items, ethnic foods commonly consumed in the Houston area, an open-ended section, and other dietary behavior questions regarding such factors as dining at restaurants and food preparation methods. From the dietary information obtained in the FFQ, total energy intake and amount consumed (g/day) for each food or beverage item were calculated based on the United States Department of Agriculture (USDA) National Nutrient Database for Standard Reference^50^. For multi-ingredient foods items not included in Standard Reference, calculations were based on the US Department of Agriculture Food and Nutrient Database for Dietary Studies^51^. Blood samples (40 mL each) were collected from the study participants for genotyping.

### Exclusions and eligibility

The exclusions and eligibility criteria for this study were previously reported^46^. For the present study, we limited the analysis to non-Hispanic whites because of the existing dietary variations between non-Hispanic whites and other racial/ethnic groups^52^,^53^ as well as insufficient statistical power for other racial/ethnic groups. Also, we further excluded those (n = 138) with outlying total energy intake. The final analysis in this study included 2139 cases and 2163 controls.

### Dietary pattern analysis

The details of dietary pattern analysis were reported in our previous study^20^. Briefly, 117 out of 159 food and beverage items in the FFQ were included in the final dietary pattern analysis. We excluded foods with low frequency of consumption (<5%) in our study population. Also, several dietary items were grouped into predefined food groups according to current US Department of Agriculture food-group guidelines. For the remaining 117 dietary items, the daily intake was log-transformed and then energy-adjusted using the residual method^54^.

We conducted an exploratory factor analysis with the FACTOR command in Stata 13.0 (StataCorp LP, College Station, Texas) to reduce the number of dietary items into a small number of factors, and the factors were then rotated using a varimax rotation, an orthogonal rotation procedure. The Kaiser-Meyer-Olkin statistic was equal to 0.85, suggesting a “meritorious” sampling adequacy (relative to the number of dietary items) for conducting dietary pattern analysis. The number of factors that best represented the data was chosen on the basis of eigenvalues greater than one, identification of a break point in the scree plot, interpretability, and our previous dietary pattern analysis on renal cell carcinoma^20^. We identified three factors that best represented the dietary input data, and these three dietary patterns were identical to the three patterns identified in our previous study of renal cell carcinoma^20^. The final analysis was restricted only to the three chosen factors. For each participant, a factor score was computed for each of the three identified factors to indicate levels of adherence to one dietary pattern with higher scores indicating higher adherence. Factor scores were categorized into quintiles based on the sex-specific distribution in the control group.

### Selection of SNPs

We initially selected nine common (minor allele frequency >5%) single-nucleotide polymorphisms (SNPs, rs1051730, rs3117582, rs8034191, rs2808630, rs7626795, rs401681, rs402710, rs8042374, and rs17879961) that were identified in previous GWA studies among individuals of European ancestry^11^,^12^,^13^,^14^,^15^,^16^. Among them, seven SNPs (except rs8042374 and rs17879961) were directly genotyped for a subset of 855 cases and 1036 controls in our previous GWAS, and rs6495309 was selected as a proxy for rs8042374 because they are in high linkage disequilibrium (LD r^2^ = 0.95). Genotyping procedures were previously reported in details^11^. One SNP (rs401681) was not included because it was not in Hardy-Weinberg equilibrium (*P* = 0.02). Furthermore, rs1051730 and rs8034191 are highly correlated (LD r^2^ = 0.85), so only rs1051730 was included in the analysis, leaving six SNPs (rs1051730, rs3117582, rs2808630, rs7626795, rs402710, and rs6495309) included in the final analysis.

### Statistical Analysis

Baseline characteristics of cases and controls were compared using the Student’s t-test for continuous variables and Chi-squared test for categorical variables. Unconditional multivariate logistic regression was used to calculate odds ratios (OR) with 95% confidence intervals (95% CI) after adjustment of potential confounders based on *a priori* knowledge. Patients with missing covariates were not included in the multivariate analyses. Trend tests were conducted by including the quintiles of the dietary pattern factor score as an ordinal variable. We also assessed whether smoking status and GWAS-identified lung cancer susceptibility loci modified the associations between dietary patterns and lung cancer risk. Multiplicative interaction was assessed by the likelihood ratio test. All statistical analyses were performed in Stata 13.0 (StataCorp LP, College Station, Texas). A *P* value < 0.05 (two-sided) was considered statistically significant.

## Additional Information

**How to cite this article**: Tu, H. *et al*. Different dietary patterns and reduction of lung cancer risk: A large case-control study in the U.S. *Sci. Rep*. **6**, 26760; doi: 10.1038/srep26760 (2016).

## Supplementary Material

Supplementary Information

## Figures and Tables

**Table 1:** Rotated factor loadings for the top 10 food items/groups contributing to each factor.

Foods items	Fruits and vegetables (Factor 1)	American/Western (Factor 2)	Tex-Mex (Factor 3)
Deep yellow vegetables	0.67		
Cruciferous vegetables	0.60		
Dark leafy green vegetables	0.59		
Apples, pears	0.56		
Melons	0.50		
Tomatoes	0.47		
Grapes	0.47		
Strawberries	0.47		
Bananas	0.43		
Peaches	0.42		
Hamburgers, cheeseburgers		0.49	
French fries, fried potatoes		0.48	
Fried chicken		0.47	
Biscuits, rolls		0.44	
Chicken fried steak		0.44	
Gravies		0.42	
Pork chops, pork roasts, dinner ham		0.41	
Bacons		0.39	
Sausage, chorizo		0.39	
Cheese dishes		0.39	
Salsa			0.57
Enchiladas			0.54
Spanish rice			0.54
Refried beans, pinto beans			0.48
Green chilis, jalapenos, serrano peppers			0.48
Avocado, guacamole			0.47
Flour tortillas			0.46
Soft tacos			0.45
Flautas, crispy tacos			0.44
Corn tortillas			0.38

**Table 2:** Selected host characteristics of non-small cell lung cancer cases and healthy controls.

Characteristics	Cases N = 2,139	Controls N = 2,163	*P* value^a^
Age, mean (SD), yrs.	61.8 (10.4)	61.9 (9.7)	0.82
Sex, No. (%)			
Men	1,103 (51.6)	1,127 (52.1)	
Women	1,036 (48.4)	1,036 (47.9)	0.72
Education, No. (%)			
High school and below	784 (36.8)	492 (22.8)	
Some college	709 (33.2)	797 (36.9)	
Completed college and above	640 (30.0)	870 (40.3)	<0.001
Smoking			
Status, No. (%)			
Never smokers	382 (17.9)	428 (19.8)	
Former smokers	1,009 (47.2)	965 (44.6)	
Current smokers	748 (35.0)	770 (35.6)	0.15
Pack-year, mean (SD)			
Former smokers	45.6 (31.1)	40.9 (31.6)	<0.001
Current smokers	55.4 (30.4)	45.6 (28.6)	<0.001
Family history of lung cancer among 1° relatives, No. (%)			
No	1,632 (76.6)	1,802 (83.6)	
Yes	499 (23.4)	353 (16.4)	<0.001
Body mass index (kg/m^2^), No. (%)			
<25 (normal or underweight)	775 (38.9)	724 (33.7)	
25–29.99 (overweight)	783 (39.3)	837 (39.0)	
>= 30 (obese)	435 (21.8)	586 (27.3)	<0.001
Physical activity level (METs/week), No. (%)			
<12 (low)	860 (40.7)	770 (35.7)	
12–30 (medium)	854 (40.4)	846 (39.2)	
>30 (high)	400 (18.9)	542 (25.1)	<0.001
Total nutrient intake, mean (SD)			
Energy, kcal/d	2011.1 (824.7)	2036.9 (741.1)	0.28
Carbohydrate, g/d	253.0 (111.7)	258.2 (100.5)	0.11
Protein, g/d	77.7 (33.4)	80.6 (30.8)	0.003
Fat, g/d	74.2 (35.5)	74.6 (32.7)	0.67

^a^*P* value by Student’s t-test for continuous variables and Chi-squared test for categorical variables.

**Table 3:** Associations between dietary patterns (quintile) and non-small cell lung cancer risk.

Dietary patterns	Cases N	Controls N	Age- and sex-adjusted OR (95% CI)	*P* value	Multivariable-adjusted^a^ OR (95% CI)	*P* value
Fruits and vegetables						
Quintile 1 (low)	578	434	Reference	N/A	Reference	N/A
Quintile 2	458	431	0.79 (0.66–0.95)	0.01	0.94 (0.77–1.14)	0.52
Quintile 3	415	434	0.71 (0.59–0.85)	<0.001	0.85 (0.69–1.03)	0.10
Quintile 4	381	432	0.65 (0.54–0.79)	<0.001	0.84 (0.68–1.03)	0.09
Quintile 5 (high)	307	432	0.52 (0.43–0.63)	<0.001	0.68 (0.55–0.85)	0.001
*P* for trend			<0.001		0.001	
American/Western						
Quintile 1 (low)	367	434	Reference	N/A	Reference	N/A
Quintile 2	334	432	0.92 (0.75–1.12)	0.39	1.02 (0.83–1.26)	0.86
Quintile 3	396	433	1.08 (0.89–1.32)	0.42	1.10 (0.89–1.35)	0.37
Quintile 4	482	432	1.32 (1.09–1.60)	0.004	1.33 (1.09–1.64)	0.006
Quintile 5 (high)	560	432	1.54 (1.28–1.86)	<0.001	1.45 (1.18–1.78)	<0.001
*P* for trend			<0.001		<0.001	
Tex-Mex						
Quintile 1 (low)	689	434	Reference	N/A	Reference	N/A
Quintile 2	472	432	0.68 (0.57–0.81)	<0.001	0.66 (0.54–0.79)	<0.001
Quintile 3	382	433	0.54 (0.45–0.65)	<0.001	0.58 (0.48–0.70)	<0.001
Quintile 4	308	432	0.43 (0.36–0.52)	<0.001	0.48 (0.40–0.59)	<0.001
Quintile 5 (high)	288	432	0.40 (0.33–0.48)	<0.001	0.45 (0.37–0.56)	<0.001
*P* for trend			<0.001		<0.001	

^a^Adjusted for age, sex, education, smoking status, pack-years, family history of lung cancer among 1° relatives, body mass index, physical activity, and total energy intake.

Abbreviations: OR, odds ratio; CI, confidence interval.

**Table 4:** Associations between dietary patterns (quintile) and non-small cell lung cancer risk by histological types.

Dietary patterns	Controls N	Adenocarcinoma		Squamous cell carcinoma		Other non-small cell carcinoma	
		N	OR^a^ (95% CI)	N	OR^a^ (95% CI)	N	OR^a^ (95% CI)
Fruits and vegetables							
Quintile 1 (low)	434	291	Reference	143	Reference	101	Reference
Quintile 2	431	233	0.88 (0.69–1.11)	101	0.87 (0.63–1.20)	86	1.23 (0.87–1.75)
Quintile 3	434	245	0.92 (0.72–1.16)	92	0.78 (0.56–1.08)	54	0.74 (0.50–1.10)
Quintile 4	432	219	0.86 (0.67–1.10)	76	0.72 (0.50–1.02)	59	0.94 (0.64–1.40)
Quintile 5 (high)	432	204	0.76 (0.59–0.98)	50	0.49 (0.33–0.74)	36	0.61 (0.39–0.97)
*P* for trend			0.06		0.001		0.02
American/Western							
Quintile 1 (low)	434	230	Reference	61	Reference	53	Reference
Quintile 2	432	198	0.99 (0.77–1.27)	65	1.08 (0.72–1.62)	69	0.80 (0.50–1.29)
Quintile 3	433	228	1.09 (0.85–1.39)	93	1.37 (0.94–2.02)	77	1.02 (0.65–1.58)
Quintile 4	432	241	1.15 (0.90–1.47)	115	1.61 (1.11–2.34)	132	1.58 (1.05–2.38)
Quintile 5 (high)	432	295	1.37 (1.07–1.75)	128	1.59 (1.09–2.32)	162	1.81 (1.20–2.71)
*P* for trend			0.005		0.002		<0.001
Tex-Mex							
Quintile 1 (low)	434	362	Reference	160	Reference	183	Reference
Quintile 2	432	278	0.72 (0.58–0.90)	94	0.58 (0.42–0.80)	121	0.56 (0.39–0.80)
Quintile 3	433	208	0.55 (0.44–0.69)	88	0.66 (0.48–0.90)	81	0.51 (0.35–0.73)
Quintile 4	432	172	0.48 (0.38–0.61)	59	0.46 (0.32-0.66)	67	0.45 (0.30–0.66)
Quintile 5 (high)	432	172	0.45 (0.35–0.58)	61	0.54 (0.38–0.77)	41	0.34 (0.22–0.52)
*P* for trend			<0.001		<0.001		<0.001

^a^Adjusted for age, sex, education, smoking status, pack-years, family history of lung cancer among 1° relatives, body mass index, physical activity, and total energy intake.

Abbreviations: OR, odds ratio; CI, confidence interval.

**Table 5:** Associations between dietary patterns (quintile) and non-small cell lung cancer risk by smoking status.

Dietary Patterns	Never smokers		Former smokers		Current smokers		*P* for interaction
	Cases/controls	OR^a^ (95% CI)	Cases/controls	OR^b^ (95% CI)	Cases/controls	OR^b^ (95% CI)	
Fruits and vegetables							
Quintile 1 (low)	73/67	Reference	205/130	Reference	300/237	Reference	0.03
Quintile 2	65/86	0.87 (0.53–1.41)	198/171	0.93 (0.68–1.28)	195/174	1.01 (0.76–1.34)	
Quintile 3	75/82	0.97 (0.60–1.57)	208/198	0.85 (0.62–1.16)	132/154	0.83 (0.61–1.13)	
Quintile 4	80/89	1.06 (0.66–1.70)	219/215	0.95 (0.69-1.29)	82/128	0.60 (0.42–0.85)	
Quintile 5 (high)	89/104	0.99 (0.62–1.58)	179/251	0.66 (0.48–0.90)	39/77	0.54 (0.34–0.84)	
*P* for trend		0.73		0.02		<0.001	
American/Western							
Quintile 1 (low)	67/93	Reference	218/244	Reference	82/ 97	Reference	0.44
Quintile 2	69/101	1.15 (0.72–1.82)	171/196	1.04 (0.78–1.38)	94/135	0.90 (0.59–1.37)	
Quintile 3	76/87	1.35 (0.84–2.15)	192/197	1.08 (0.81–1.44)	128/149	0.97 (0.65–1.46)	
Quintile 4	79/83	1.46 (0.91–2.32)	214/171	1.31 (0.98–1.75)	189/178	1.25 (0.85–1.83)	
Quintile 5 (high)	91/64	2.01 (1.25–3.24)	214/157	1.39 (1.03–1.87)	255/211	1.26 (0.87–1.83)	
*P* for trend		0.002		0.01		0.03	
Tex-Mex							
Quintile 1 (low)	115/91	Reference	333/198	Reference	241/145	Reference	0.87
Quintile 2	73/68	0.82 (0.52–1.29)	232/216	0.61 (0.47–0.80)	167/148	0.64 (0.47–0.89)	
Quintile 3	69/84	0.64 (0.42–1.00)	182/182	0.59 (0.44–0.78)	131/167	0.54 (0.39–0.75)	
Quintile 4	64/89	0.59 (0.38–0.92)	135/187	0.45 (0.33–0.60)	109/156	0.48 (0.34–0.67)	
Quintile 5 (high)	61/96	0.50 (0.32–0.78)	127/182	0.44 (0.33–0.60)	100/154	0.45 (0.32–0.63)	
*P* for trend		0.001		<0.001		<0.001	

^a^Adjusted for age, sex, education, family history of lung cancer among 1° relatives, body mass index, physical activity, and total energy intake.

^b^Adjusted for age, sex, education, pack-years, family history of lung cancer among 1° relatives, body mass index, physical activity, and total energy intake.

Abbreviations: OR, odds ratio; CI, confidence interval.

**Table 6:** Associations between dietary patterns (quintile) and non-small cell lung cancer risk by genotype at a lung cancer susceptibility locus (*CRP* rs2808630).

Dietary patterns	Cases/controls	OR^a^ (95% CI)	Cases/controls	OR^a^ (95% CI)	*P* for interaction
	rs2808630: AA		rs2808630: AG/GG		
Fruits and vegetables					
Quintile 1 (low)	114/97	Reference	115/98	Reference	0.03
Quintile 2	82/98	0.94 (0.62–1.43)	104/105	0.97 (0.65–1.46)	
Quintile 3	87/103	0.89 (0.59–1.36)	88/99	0.95 (0.62–1.44)	
Quintile 4	69/118	0.69 (0.45–1.06)	88/101	1.01 (0.66–1.54)	
Quintile 5 (high)	38/116	0.42 (0.26–0.69)	69/101	0.82 (0.53–1.28)	
*P* for trend		0.001		0.50	
American/Western					
Quintile 1 (low)	75/95	Reference	72/108	Reference	0.02
Quintile 2	56/91	0.84 (0.52–1.35)	65/94	1.17 (0.74–1.86)	
Quintile 3	63/112	0.67 (0.42-1.06)	79/102	1.22 (0.78–1.90)	
Quintile 4	102/124	0.99 (0.64–1.52)	95/96	1.53 (0.99–2.37)	
Quintile 5 (high)	94/110	0.91 (0.59–1.43)	153/104	1.93 (1.27–2.93)	
*P* for trend		0.93		0.001	
Tex–Mex					
Quintile 1 (low)	118/104	Reference	171/118	Reference	0.27
Quintile 2	96/123	0.64 (0.43–0.95)	103/106	0.63 (0.43–0.92)	
Quintile 3	61/109	0.53 (0.35–0.82)	74/95	0.54 (0.36–0.81)	
Quintile 4	62/100	0.60 (0.39–0.92)	63/99	0.45 (0.30–0.68)	
Quintile 5 (high)	53/96	0.54 (0.35–0.85)	53/86	0.44 (0.28–0.68)	
*P* for trend		0.006		<0.001	

^a^Adjusted for age, sex, education, smoking status, pack-years, family history of lung cancer among 1° relatives, body mass index, physical activity, and total energy intake.

Abbreviations: *CRP*, C-reactive protein; OR, odds ratio; CI, confidence interval.
