# Supplementary material for: Different dietary patterns and reduction of lung cancer risk: A large case-control study in the U.S
Source: Sci Rep. 2016 May 27;6:26760. doi: 10.1038/srep26760 (PMC4882747; doi:10.1038/srep26760)
Supplement: Supplementary Information [file srep26760-s1.pdf]

Different dietary patterns and reduction of lung cancer risk:

A large case-control study in the U.S.

Huakang Tu[1], PhD; John V. Heymach[2], MD, PhD; Chi-Pang Wen[3], MD, DrPH; Yuanqing Ye[1], PhD; Jeanne A. Pierzynski[1], MPH; Jack A. Roth[4], MD; Xifeng Wu[1*], MD, PhD

Departments of [1]Epidemiology, [2]Thoracic/Head and Neck Medical Oncology, [4]Thoracic and Cardiovascular Surgery, The University of Texas MD Anderson Cancer Center, Houston, Texas.

[3]Institute of Population Health Science, National Health Research Institutes, Zhunan, Taiwan.

Supplementary Table 1. Spearman correlation coefficients of the factor scores for dietary patterns with nutrient intake

Nutrient intake	Fruits and vegetables pattern	American/Western pattern	Tex-Mex pattern
**Macronutrients**			
Carbohydrate,% of total energy intake	0.33	-0.25	-0.23
Fat, % of total energy intake	-0.34	0.35	0.15
Protein, % of total energy intake	0.15	0.04	0.12
**Micronutrients** [a]			
Dietary fiber, g/day	0.65	-0.32	0.04
Cholesterol, mg/day	-0.12	0.27	0.07
Sodium, mg/day	-0.004	0.19	0.26
Potassium, mg/day	0.61	-0.25	0.008
Calcium, mg/day	0.23	-0.17	-0.03
Iron, mg/day	0.31	-0.08	0.02
Magnesium, mg/day	0.56	-0.36	0.07
Vitamin A, IU/day	0.65	-0.22	-0.07
Beta-carotene, IU/day	0.64	-0.22	-0.06
Vitamin B6, mg/day	0.46	-0.21	0.03
Vitamin C, mg/day	0.59	-0.32	0.02

[a] Energy adjusted using the residual method

Supplementary Table 2. Associations between dietary patterns and non-small cell lung cancer risk by genotype at five lung cancer susceptibility loci

Dietary patterns	Cases/controls	OR[a] (95% CI)	Cases/controls	OR[a] (95% CI)	*P* for interaction
	**rs1051730: GG**		**rs1051730: GA/AA**		
Fruits and vegetables					
Quintile 1 (low)	76/85	Reference	154/110	Reference	0.54
Quintile 2	69/96	1.00 (0.63-1.58)	117/107	0.95 (0.65-1.38)	
Quintile 3	65/88	1.13 (0.71-1.82)	110/114	0.80 (0.55-1.17)	
Quintile 4	59/104	0.87 (0.54-1.40)	97/115	0.82 (0.55-1.21)	
Quintile 5 (high)	40/93	0.69 (0.41-1.17)	67/124	0.57 (0.37-0.86)	
P for trend		0.16		0.009	
American/Western					
Quintile 1 (low)	55/101	Reference	92/102	Reference	0.46
Quintile 2	47/91	0.99 (0.60-1.65)	74/94	1.00 (0.65-1.55)	
Quintile 3	45/96	0.78 (0.47-1.29)	97/118	0.99 (0.65-1.49)	
Quintile 4	75/87	1.54 (0.96-2.49)	122/133	1.02 (0.69-1.53)	
Quintile 5 (high)	87/91	1.40 (0.88-2.25)	160/123	1.32 (0.89-1.96)	
P for trend		0.03		0.17	
Tex-Mex					
Quintile 1 (low)	108/119	Reference	181/103	Reference	0.52
Quintile 2	70/101	0.77 (0.50-1.17)	129/128	0.51 (0.35-0.73)	
Quintile 3	51/86	0.71 (0.45-1.11)	83/118	0.41 (0.28-0.60)	
Quintile 4	49/93	0.58 (0.37-0.92)	77/106	0.45 (0.30-0.67)	
Quintile 5 (high)	31/67	0.53 (0.31-0.89)	75/115	0.40 (0.27-0.60)	
P for trend		0.004		<0.001	
	rs3117582: AA		rs3117582: AC/CC		
Fruits and vegetables					
Quintile 1 (low)	185/151	Reference	45/44	Reference	0.13
Quintile 2	149/156	0.95 (0.68-1.32)	37/47	1.00 (0.53-1.87)	
Quintile 3	133/150	0.92 (0.66-1.29)	42/52	0.93 (0.50-1.73)	
Quintile 4	120/176	0.78 (0.55-1.10)	37/43	1.08 (0.57-2.05)	
Quintile 5 (high)	81/180	0.55 (0.38-0.80)	26/36	0.95 (0.48-1.91)	
P for trend		0.002		0.99	
American/Western					
Quintile 1 (low)	125/158	Reference	22/44	Reference	0.19
Quintile 2	94/151	0.90 (0.63-1.31)	27/34	1.49 (0.70-3.17)	
Quintile 3	114/161	0.95 (0.66-1.36)	28/53	0.85 (0.41-1.76)	
Quintile 4	147/175	1.09 (0.77-1.53)	50/45	1.94 (0.97-3.87)	
Quintile 5 (high)	188/168	1.25 (0.89-1.77)	60/46	2.01 (1.01-3.98)	
P for trend		0.10		0.02	
Tex-American					

Quintile 1 (low)	227/181	Reference	62/40	Reference	0.60
Quintile 2	163/180	0.67 (0.50-0.91)	36/49	0.45 (0.24-0.82)	
Quintile 3	101/163	0.51 (0.36-0.71)	34/41	0.60 (0.32-1.13)	
Quintile 4	98/148	0.56 (0.40-0.78)	28/51	0.38 (0.20-0.71)	
Quintile 5 (high)	79/141	0.50 (0.35-0.72)	27/41	0.39 (0.20-0.75)	
P for trend		<0.001		0.003	
	rs7626795: AA		rs7626795: AG/GG		
Fruits and vegetables					
Quintile 1 (low)	183/145	Reference	47/50	Reference	0.49
Quintile 2	146/139	1.01 (0.72-1.42)	40/64	0.84 (0.46-1.51)	
Quintile 3	142/143	0.99 (0.70-1.39)	33/59	0.73 (0.39-1.34)	
Quintile 4	124/154	0.87 (0.61-1.23)	33/65	0.75 (0.41-1.39)	
Quintile 5 (high)	76/158	0.57 (0.39-0.84)	31/58	0.79 (0.42-1.47)	
P for trend		0.007		0.40	
American/Western					
Quintile 1 (low)	117/147	Reference	30/56	Reference	0.66
Quintile 2	97/133	1.01 (0.69-1.47)	24/52	0.96 (0.49-1.91)	
Quintile 3	105/145	0.91 (0.63-1.32)	37/69	1.00 (0.54-1.88)	
Quintile 4	156/161	1.19 (0.83-1.69)	41/58	1.35 (0.72-2.54)	
Quintile 5 (high)	196/153	1.36 (0.95-1.93)	52/61	1.49 (0.81-2.73)	
P for trend		0.04		0.10	
Tex-American					
Quintile 1 (low)	230/154	Reference	59/68	Reference	0.24
Quintile 2	149/175	0.53 (0.39-0.73)	50/54	0.96 (0.56-1.66)	
Quintile 3	104/145	0.51 (0.36-0.72)	31/59	0.59 (0.33-1.04)	
Quintile 4	100/139	0.51 (0.36-0.72)	26/59	0.53 (0.29-0.96)	
Quintile 5 (high)	88/126	0.51 (0.35-0.73)	18/56	0.36 (0.19-0.70)	
P for trend		<0.001		<0.001	
	rs402710: GG		rs402710: GA/AA		
Fruits and vegetables					
Quintile 1 (low)	105/73	Reference	92/87	Reference	0.54
Quintile 2	80/87	0.82 (0.52-1.28)	74/90	0.94 (0.60-1.48)	
Quintile 3	76/91	0.74 (0.47-1.17)	70/73	1.14 (0.71-1.81)	
Quintile 4	68/90	0.78 (0.49-1.25)	68/97	0.90 (0.57-1.41)	
Quintile 5 (high)	48/90	0.59 (0.36-0.97)	42/101	0.57 (0.35-0.94)	
P for trend		0.05		0.06	
American/Western					
Quintile 1 (low)	74/89	Reference	49/84	Reference	0.85
Quintile 2	47/77	0.75 (0.45-1.24)	55/81	1.33 (0.79-2.24)	
Quintile 3	61/86	0.82 (0.51-1.33)	64/99	1.09 (0.66-1.79)	
Quintile 4	94/97	1.10 (0.70-1.72)	77/90	1.45 (0.88-2.38)	
Quintile 5 (high)	101/82	1.18 (0.74-1.86)	101/94	1.58 (0.97-2.57)	

P for trend		0.18		0.07	
Tex-American					
Quintile 1 (low)	124/81	Reference	112/101	Reference	0.55
Quintile 2	86/95	0.54 (0.35-0.83)	85/101	0.73 (0.48-1.11)	
Quintile 3	60/85	0.46 (0.29-0.72)	55/89	0.58 (0.37-0.92)	
Quintile 4	63/88	0.47 (0.30-0.75)	50/83	0.59 (0.37-0.93)	
Quintile 5 (high)	44/82	0.37 (0.23-0.60)	44/74	0.58 (0.36-0.94)	
P for trend		<0.001		0.008	
	**rs6495309: GG**		**rs6495309: GA/AA**		
Fruits and vegetables					
Quintile 1 (low)	148/116	Reference	82/78	Reference	0.86
Quintile 2	125/125	0.92 (0.64-1.33)	61/78	0.99 (0.61-1.61)	
Quintile 3	119/124	0.89 (0.61-1.30)	56/78	0.94 (0.57-1.53)	
Quintile 4	107/136	0.82 (0.56-1.20)	50/83	0.83 (0.51-1.38)	
Quintile 5 (high)	74/141	0.59 (0.39-0.88)	33/76	0.64 (0.37-1.11)	
P for trend		0.01		0.10	
American/Western					
Quintile 1 (low)	95/126	Reference	52/77	Reference	0.64
Quintile 2	82/120	0.97 (0.65-1.46)	39/65	1.04 (0.59-1.84)	
Quintile 3	103/131	1.02 (0.69-1.52)	39/82	0.75 (0.43-1.29)	
Quintile 4	134/148	1.16 (0.80-1.70)	63/72	1.35 (0.80-2.27)	
Quintile 5 (high)	159/117	1.52 (1.03-2.23)	89/97	1.22 (0.75-2.00)	
P for trend		0.02		0.22	
Tex-Mex					
Quintile 1 (low)	189/129	Reference	100/92	Reference	0.92
Quintile 2	124/142	0.57 (0.40-0.80)	75/87	0.71 (0.46-1.11)	
Quintile 3	100/125	0.58 (0.41-0.83)	35/79	0.41 (0.25-0.68)	
Quintile 4	88/124	0.50 (0.35-0.73)	38/75	0.50 (0.30-0.83)	
Quintile 5 (high)	72/122	0.44 (0.30-0.65)	34/60	0.53 (0.31-0.89)	
P for trend		<0.001		0.001	

[a] Adjusted for age, sex, education, smoking status, pack-years, family history of lung cancer among 1° relatives, body mass index, physical activity, and total energy intake.
Abbreviations: OR, odds ratio; CI, confidence interval
